# Association between Chinese visceral adiposity index and metabolic-associated fatty liver disease in Chinese adults with type 2 diabetes mellitus

**DOI:** 10.3389/fendo.2022.935980

**Published:** 2022-08-01

**Authors:** Min Tang, Xiao-Hui Wei, Han Cao, Qin Zhen, Fang Liu, Yu-Fan Wang, Neng-Guang Fan, Yong-De Peng

**Affiliations:** ^1^ Department of Endocrinology and Metabolism, Shanghai General Hospital, Shanghai Jiao Tong University School of Medicine, Shanghai, China; ^2^ Shanghai General Hospital of Nanjing Medical University, Shanghai, China; ^3^ Department of Endocrinology, Songjiang District Central Hospital, Shanghai, China

**Keywords:** Chinese visceral adiposity index, type 2 diabetes mellitus, visceral adiposity, metabolic-associated fatty liver disease, non-alcoholic fatty liver disease

## Abstract

**Objective:**

The purpose of the study was to determine the correlation of the Chinese visceral adiposity index (CVAI) with metabolic-associated fatty liver disease (MAFLD) in Chinese adults with type 2 diabetes mellitus (T2DM).

**Materials/methods:**

In this cross-sectional study, data on sociodemographic characteristics, laboratory test results, coexisting diseases, and medical therapy were collected and analyzed. Multivariate logistic regression analyses were used to examine the correlation between CVAI and MAFLD. In order to investigate the correlation between CVAI on a continuous scale and MAFLD, a restricted cubic spline (RCS) was used.

**Results:**

A total of 679 participants were included in this study. There were 251 female participants and 428 male participants, with a median age of 55 years. In the multivariate logistic regression model, diastolic blood pressure, duration of diabetes, glycated hemoglobin, hemoglobin, alanine transaminase, aspartate aminotransferase, gamma -glutamyl transferase, albumin, blood urea nitrogen, total cholesterol, low-density lipoprotein cholesterol, statin use and metformin use were adjusted, and an evident increase in the odds ratios of MAFLD from the lowest to the highest CVAI quartile was found (*P* value for trend < 0.001). Moreover, the RCS curves revealed a positive correlation between CVAI and MAFLD.

**Conclusions:**

The CVAI is positively correlated with MAFLD and may be an indicator with diagnostic value for MAFLD in clinical practice in type 2 diabetic patients.

## Introduction

The prevalence of diabetes mellitus (DM) is growing rapidly, with approximately 463 million people having DM in 2019, and this number will increase to 578 million in 2030, which leads to one in nine deaths among adults (1, 2). Among patients with type 2 DM (T2DM), the incidence of non-alcoholic fatty liver disease (NAFLD) is evidently increasing (3–5). Patients with T2DM with comorbid NAFLD tend to develop severe liver diseases such as nonalcoholic steatohepatitis, advanced fibrosis, and cirrhosis (3–5). Data from previous studies have suggested that about 60–70% of patients with T2DM had NAFLD, and approximately 15% developed advanced liver fibrosis (6, 7). NAFLD was renamed as metabolic-associated fatty liver disease (MAFLD) in 2020 (8). Published studies indicated that the proportion of MAFLD population was 51% among obese population (9), and that approximately 20% of patients with MAFLD could develop nonalcoholic steatohepatitis (5). Thus, screening for MAFLD is necessary for early treatment, especially since liver biopsy, an invasive tool for the diagnosis of MAFLD, is not always available for use in clinical practice (10).

Accumulated evidence has revealed that adipose tissue distribution was crucial to the development of metabolic diseases and all-cause mortality (11–13). Some examinations can identify abdominal adiposity, including dual-energy X-ray absorptiometry, computed tomography, and magnetic resonance imaging (14); however, these examinations are unsuitable for epidemiologic studies and routine clinical practices. A published study has indicated that the Chinese visceral adiposity index (CVAI) is a reliable tool for assessing visceral adiposity and can predict the prevalence of metabolic disorders in Chinese population (15). CVAI has been reported to better predict prediabetes and T2DM than body mass index (BMI), waist circumference (WC), waist-to-height ratio, and visceral adiposity index in Chinese population (16–18). A previous study has revealed that CVAI is correlated with the risk of NAFLD in Chinese adults (19). However, evidence on the association between the CVAI and MAFLD in patients with T2DM is limited.

The purpose of this cross-sectional study was to examine the relation of CVAI with MAFLD in Chinese adults with T2DM.

## Materials and methods

### Study design

We recruited 679 participants who met the criteria of this cross-sectional study from the National Metabolic Management Center (MMC) of Shanghai General Hospital from April 2017 to September 2021. Ethics committee approval was obtained from Shanghai General Hospital, Shanghai Jiao Tong University School of Medicine for this study.

### Study population

All the participants’ information in our study were based on the samples of MMC, and 679 participants with T2DM were enrolled in the study. Our study included participants who met the criteria for T2DM diagnosis as prescribed by the World Health Organization criteria of 1999 (20) and were at least 18 years of age. The study excluded patients with pregnancy, malignant tumor, acute infection, incomplete anthropometric data, lack of liver ultrasound data.

### Patient data collection

A standard questionnaire was made for trained staff to gather information on sociodemographic characteristics and previous medical history, including name, sex, age, educational attainment, lifestyle factors, laboratory tests, coexisting diseases, and medical therapy. The weight and height of the participants were measured using a meter (OMRON HNH-318; OMRON Corporation, Japan). WC was assessed by trained staff according to standard protocols. Blood pressure (BP) was evaluated using an automated electronic BP meter (OMRON HEM-7117; OMRON Corporation, Japan). The abdominal ultrasonograpy of all participants was assessed by professional ultrasonographers.

After participants with overnight fasting at least 10 hours, venous blood samples were gathered from them. Leukocyte count and platelet count as well as hemoglobin and high-sensitivity C-reactive protein (hs-CRP) levels were measured using an automatic hematology analyzer (BC5800, Mindray Co., Shenzhen, China). All biochemical indicators, including alanine aminotransferase (ALT), aspartate aminotransferase (AST), gamma-glutamyl transferase (GGT), albumin, blood urea nitrogen (BUN), serum creatinine, fasting plasma glucose, triglycerides, total cholesterol (TC), high-density lipoprotein cholesterol, and low-density lipoprotein cholesterol (LDL-C) levels, were determined using an automatic biochemistry analyzer (ADVIA 2400, SIEMENS AG, Germany). Glycated hemoglobin (HbA1c) levels were measured by high-performance liquid chromatography using an automatic HbA1c analyzer (TOSOH HLC-723G8, TOSOH Corporation, Japan).

### Definition of variables

The diagnosis criterion of hypertension was systolic blood pressure ≥ 140 mmHg and/or diastolic blood pressure (DBP) ≥ 90 mmHg, and the examination was with repetition (21), or with a medical history of hypertension. The diagnosis of fatty liver was used by liver ultrasound (22, 23). The diagnosis of MAFLD was in accordance with published study (24). The BMI of participants was calculated as the weight in kilograms divided by the square of their height in meters, and CVAI was calculated in accordance with the formula described in a previous study (25).

### Statistical analyses

Data are showed as numbers, mean ± standard deviation or medians (interquartile ranges). The Mann-Whitney U test or T test was performed to compare continuous variables between the two groups. Categorical variables were analyzed by chi-square test to compare differences between two groups. A two-sided P-value < 0.05 between the different groups was considered significant. All statistical analyses were performed using IBM SPSS (version 25.0; IBM Corporation, USA) and the R statistical software (version 4.1.3, http://www.Rproject.org).

In our study, we used multivariate logistic regression models to determine the relationship between CVAI and MAFLD, and to estimate odds ratios (ORs) and 95% confidence intervals (CIs). We adjusted DBP and duration of diabetes in model 1. And HbA1c, hemoglobin, ALT, AST, GGT, albumin, BUN were further adjusted in model 2. Model 3 was further adjusted for TC and LDL-C. Statin use and metformin use were added in Model 4.

We used a restricted cubic spline (RCS) to explore the relation of CVAI with MAFLD. In RCS, there were four knots located at 5th, 35th, 65th, and 95th percentiles. The relationship of CVAI and MAFLD in different subgroups classified by sex, age, hypertension, BMI, HbA1c and statin use as two groups were explored by multivariate logistic regression models. In addition, the interaction between the CVAI and the above subgroup variables was determined.

## Results

### Characteristics of patients

Study participants included 679 patients, including 251 female participants and 428 male participants. The median study participants’ age was 55 years. Among study participants, there were 381 participants without MAFLD and 298 participants with MAFLD. The characteristics of the participants in the two groups are summarized in **Table 1**. The difference of age, DBP, BMI, CVAI, duration of diabetes, HbA1c, ALT, AST, GGT, albumin, BUN, triglycerides, TC, high-density lipoprotein cholesterol, LDL-C, statin use and metformin use reached statistical significance between the two groups.

**Table 1:** Characteristics of Patients.

Variables	No MAFLD (n = 381; 56.11%)	MAFLD (n = 298; 43.89%)	P value
Sex (Female/Male)	134/247	117/181	0.273
Age (years)	57 (50–64)	53 (40-62)	<0.001
Diastolic blood pressure (mmHg)	73.37 ± 9.88	75.84 ± 10.48	0.002
Systolic blood pressure (mmHg)	123 (116-134)	124 (116-134)	0.632
BMI (kg/m^2^)	24.03 (22.10-25.79)	26.50 (24.49-29.10)	<0.001
Duration of diabetes (months)	121 (15-183)	61 (1-123)	<0.001
CVAI	114.60 (91.02-137.70)	137.25 (116.94-158.86)	<0.001
FPG (mmol/L)	7.00 (5.49-8.99)	7.41 (5.87-9.08)	0.053
HbA1c (%)	8.5 (7.1-10.5)	8.9 (7.5-11.0)	0.034
Hemoglobin (g/L)	139 (125-150)	144 (134-152)	<0.001
Leukocyte (×10^9^/L)	6.03 (5.11-7.25)	6.30 (5.29-7.55)	0.058
ALT (IU/L)	17 (13-25)	25 (17-40)	<0.001
AST (IU/L)	17 (14-21)	20 (16-29)	<0.001
GGT (IU/L)	19 (14-33)	30 (21-46)	<0.001
Albumin (g/L)	42.3 (39.8-45.2)	43.2 (40.7-45.4)	0.006
Blood urea nitrogen (mmol/L)	5.6 (4.4-7.4)	5.2 (4.0-6.9)	0.026
Creatinine (μmol/L)	59.0 (47.8-72.8)	59.4 (47.0-68.5)	0.232
Triglyceride (mmol/L)	1.44 (1.04-2.06)	1.94 (1.46-2.96)	<0.001
Total cholesterol (mmol/L)	4.51 (3.61-5.18)	4.78 (3.93-5.44)	0.003
HDL-C (mmol/L)	0.96 (0.82-1.17)	0.88 (0.77-0.98)	<0.001
LDL-C (mmol/L)	2.46 (1.80-3.08)	2.78 (2.08-3.36)	<0.001
Educational level (under high school/high school or above)	208/173	145/153	0.124
Hypertension (no/yes)	199/182	152/146	0.751
Statin use (no/yes)	179/202	116/182	0.036
Aspirin use (no/yes)	272/109	217/81	0.681
Metformin use (no/yes)	108/273	51/247	0.001

Data are presented as number, mean ± standard deviation, or median (interquartile range). Continuous variables use Mann-Whitney U test or T test and categorical variables use chi-squared test for comparing the characteristics of patients with MAFLD and without MAFLD. BMI, body mass index; CVAI, Chinese visceral adiposity index; FPG, fasting plasma glucose; HbA1c, glycated hemoglobin; ALT, alanine transaminase; AST, aspartate aminotransferase; GGT, gamma -glutamyl transferase; HDL-C, high-density lipoprotein cholesterol; LDL-C, low-density lipoprotein cholesterol; MAFLD, metabolic-associated fatty liver disease.

### Association between CVAI and MAFLD

**Table 2** suggested that an increased CVAI was associated with higher probability of MAFLD after adjusting for the confounders. In the multivariate regression model, when DBP, duration of diabetes, HbA1c, hemoglobin, ALT, AST, GGT, albumin, BUN, TC, LDL-C, statin use and metformin use were adjusted, an evident increase in the ORs of MAFLD from the lowest to the highest CVAI quartile was found (*P* value for trend< 0.001), and the OR (95% CI) of MAFLD was 4.739 (2.778–8.085) for the highest quartile compared to the lowest quartile.

**Table 2:** Relations of Chinese visceral adiposity index with metabolic-associated fatty liver disease in patients with type 2 diabetes mellitus.

CVAI					
	Q1 (N=170)	Q2 (N=170)	Q3 (N=170)	Q4 (N=169)	P value for trend
Model 1 - OR (95% CI)	Ref.	1.772 (1.095–2.866)	4.617 (2.820–7.556)	6.170 (3.754–10.141)	<0.001
Model 2 - OR (95% CI)	Ref.	1.724 (1.050–2.831)	4.126 (2.480–6.864)	4.985 (2.952–8.418)	<0.001
Model 3 - OR (95% CI)	Ref.	1.708 (1.037–2.813)	4.106 (2.461–6.851)	5.014 (2.956–8.505)	<0.001
Model 4 - OR (95% CI)	Ref.	1.657 (1.000–2.746)	4.202 (2.499–7.067)	4.739 (2.778–8.085)	<0.001

Model 1 was adjusted for diastolic blood pressure and duration of diabetes. Model 2 was further adjusted for glycated hemoglobin, hemoglobin, alanine transaminase, aspartate aminotransferase, gamma -glutamyl transferase, albumin, blood urea nitrogen. Model 3 was further adjusted for total cholesterol and low-density lipoprotein cholesterol. Model 4 was further adjusted for statin use and metformin use. The quartile ranges of Q1, Q2, Q3, and Q4 of CVAI were <100.66, 100.66-126.10, 126.10-148.22,>148.22, respectively. Q1 is the reference group. Multivariate logistic regression analyses were performed to estimate the ORs and corresponding 95% CIs for metabolic-associated fatty liver disease. Q, quartile; OR, odds ratio; CI, confidence interval; N, number; CVAI, Chinese visceral adiposity index.

**Figure 1:**
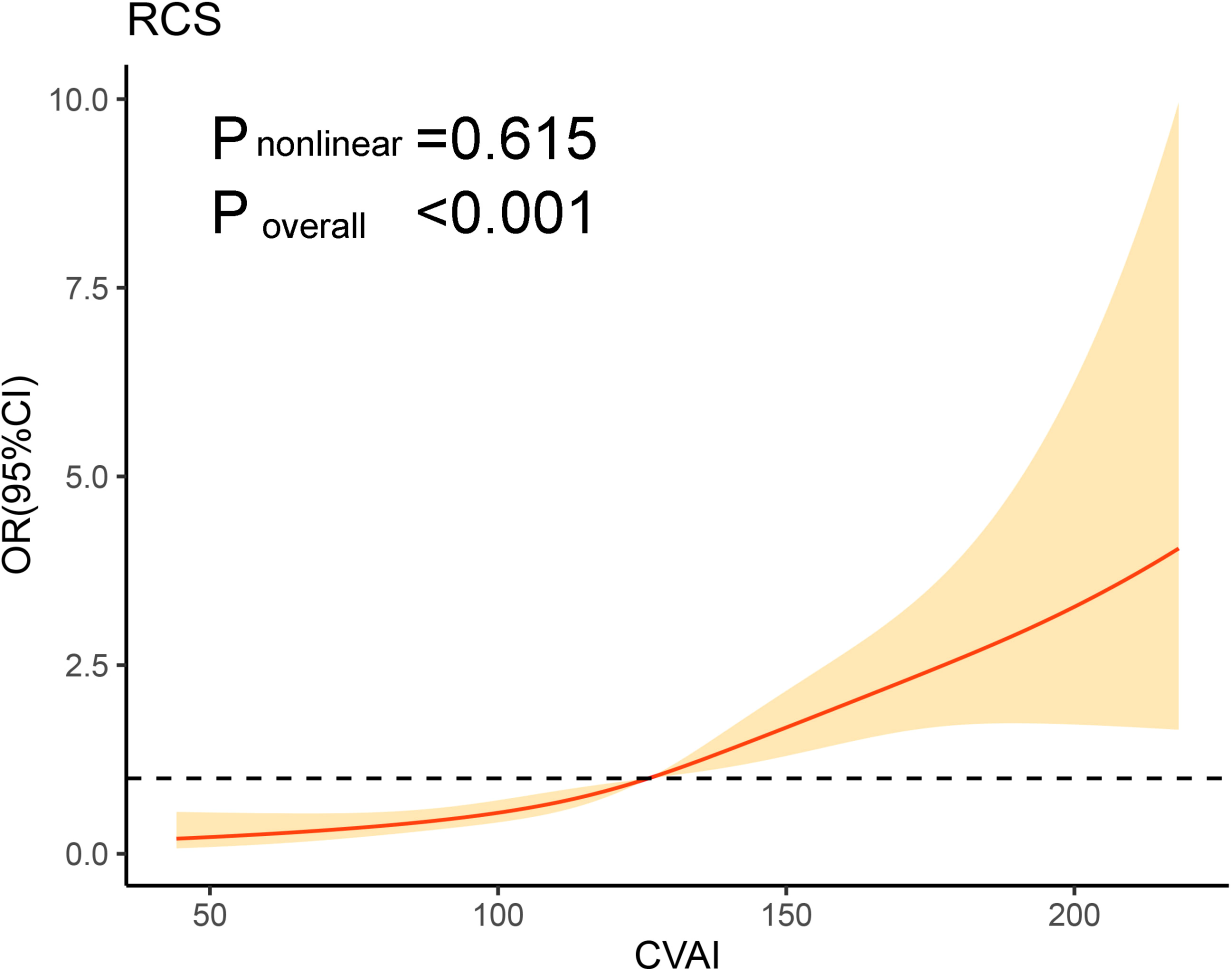
Association of CVAI on a continuous scale and metabolic-associated fatty liver disease. The solid line represents the odds ratio and the shad area represents the 95% confidence interval. Model was adjusted for diastolic blood pressure, duration of diabetes, glycated hemoglobin, hemoglobin, alanine transaminase, aspartate aminotransferase, gamma -glutamyl transferase, albumin, blood urea nitrogen, total cholesterol, low-density lipoprotein cholesterol, statin use and metformin use. CVAI, Chinese visceral adiposity index; OR, odds ratio; CI, confidence interval; RCS, restricted cubic spline.

Additionally, after adjusting for DBP, duration of diabetes, HbA1c, hemoglobin, ALT, AST, GGT, albumin, BUN, TC, LDL-C, statin use and metformin use, the RCS curves also demonstrated a positive correlation between CVAI and the presence of MAFLD (**Figure 1**).

### Subgroup analyses

**Figure 2:**
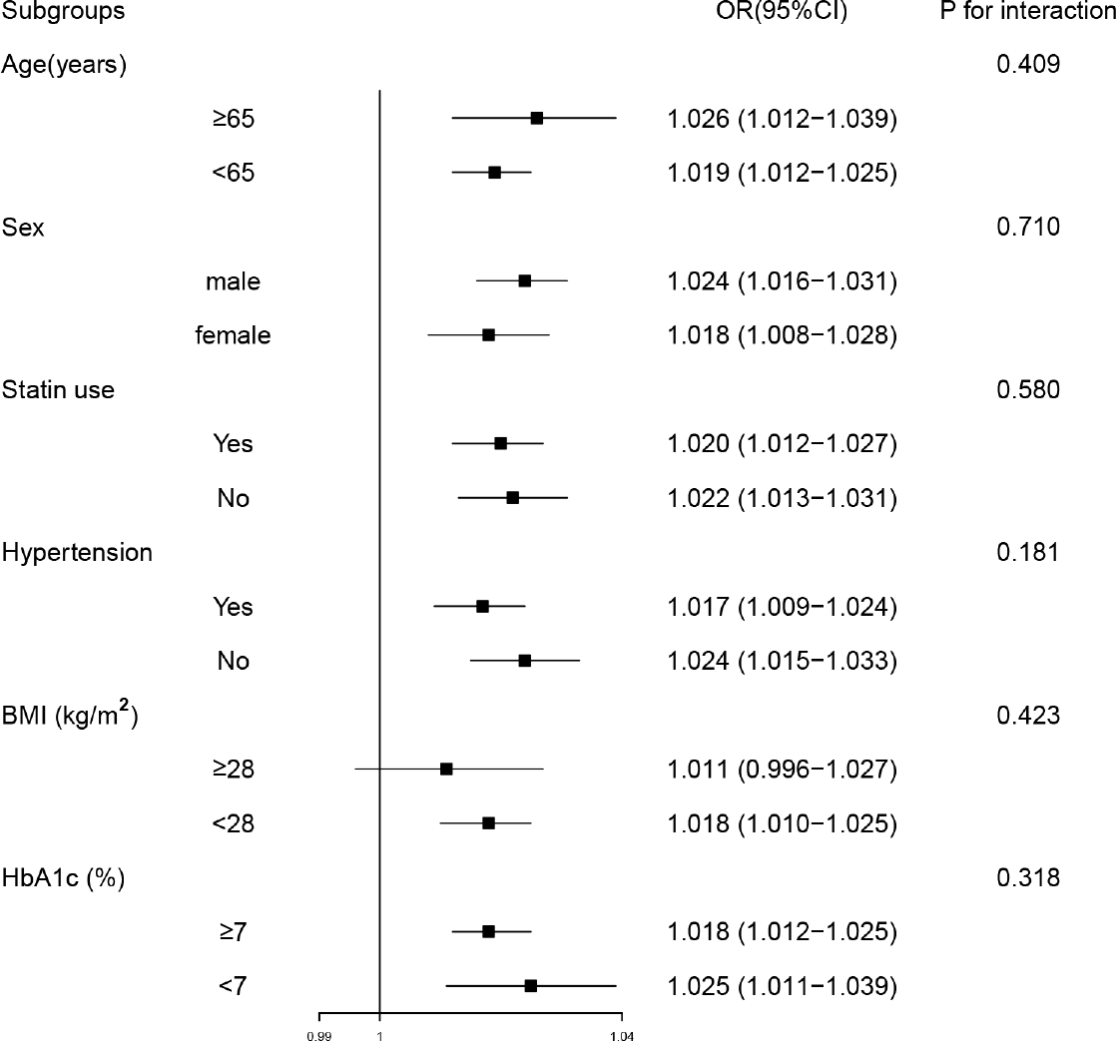
Subgroup analyses of the associations between Chinese visceral adiposity index and metabolic-associated fatty liver disease. Model was adjusted for diastolic blood pressure, duration of diabetes, glycated hemoglobin, hemoglobin, alanine transaminase, aspartate aminotransferase, gamma-glutamyl transferase, albumin, blood urea nitrogen, total cholesterol, low-density lipoprotein cholesterol, statin use and metformin use. Subgroup variable was excluded from the model. OR, odds ratio; CI, confidence interval; BMI, body mass index; HbA1c, glycated hemoglobin.

In subgroup analyses, participants were stratified by sex (male or female), age (< 65 or ≥ 65 years), hypertension (no or yes), BMI (< 28 or ≥ 28 kg/m^2^), HbA1c (< 7 or ≥ 7%), statin use (no or yes). The subgroup analyses indicated that CVAI were significantly related with MAFLD in many categories, and no significant interaction was observed between CVAI and subgroup variables (**Figure 2**).

## Discussion

In our study, we identified an association between the CVAI and MAFLD in Chinese adults with T2DM. CVAI was higher in the study participants with MAFLD than study participants without MAFLD and a significantly positive relationship was identified between the CVAI and MAFLD. Therefore, the CVAI may be an indicator with diagnostic value for MAFLD among T2DM patients in clinical practice.

It is well established that visceral obesity is related to the risk of diabetes (26). CVAI is a novel index calculated by a formula including age, BMI, WC, high-density lipoprotein cholesterol, and triglycerides levels to evaluate visceral obesity among the Chinese population (17). Several published studies have identified the optimal indicator for screening obesity-associated diseases, diabetic complications, and cardiovascular disease by comparing different anthropometric indices (16, 25, 27–29), which demonstrated that the CVAI was a reliable and optimal predictor. Similar results were observed in other published studies, which indicated that CVAI had superior performance for diagnosing DM than traditional parameters (18, 30). Moreover, a previous study has revealed that visceral adiposity confirmed by CVAI had a better predictive ability for NAFLD incidence (19). Anthropometric indicators including fatty liver index, lipid accumulation product index and hepatic steatosis index, have clinical value to diagnose and evaluate MAFLD (31). Waist-to-height ratio and the lipid accumulation product index have greater sensitivity to determine visceral fat compared to WC and BMI (32). Our results of this study found a positive correlation between MAFLD and the CVAI, which may provide scientific evidence for the use of the CVAI to diagnose MAFLD in patients with T2DM.

The accumulation of visceral fat was regarded as a key risk factor and predictor of NAFLD (33, 34). Excessive visceral adipose tissue may result in lipotoxicity, insulin resistance and the increase of pro-inflammatory mediators (35). An increase in interleukin-6, C-reactive protein, interleukin-1β, and tumor necrosis factor-α was found in the population of MAFLD (36). Because the CVAI can reflect the amount of visceral fat, we chose it as an indicator of abdominal obesity. We explored the association of the CVAI with MAFLD in patients with T2DM. However, we did not compare the predictive ability of different indicators of abdominal obesity, therefore, other studies with larger sample sizes, using different indicators are needed, to identify the superior performance of the CVAI to predict MAFLD.

Our study had several limitations. First, the study was cross-sectional, which made inferring the causal relationship between CVAI and MAFLD impossible; therefore, future prospective studies are required to investigate this causal relationship. Second, our results may be affected by the small sample size. Third, our study lacked some meaningful variables associated with obesity, such as insulin resistance index and inflammation mediators. Fourth, we lacked the parameters of liver fibrosis, thus the association between CVAI and advanced liver fibrosis could not be analyzed. Additionally, some potential confounders, such as dietary habits, physical activities, and medications, may influence MAFLD, thus interfering with the results. Moreover, in our study, the CVAI may have limited clinical applicability as the spectrum of patients selected was based upon the 1999 World Health Organization criteria. Furthermore, a positive relationship was identified in the Chinese population, and further studies that include other ethnic groups should be conducted.

In conclusion, CVAI was positively related with MAFLD. Therefore, the CVAI may be an be an indicator with diagnostic value for MAFLD among T2DM patients in clinical practice.

## Data availability statement

The raw data supporting the conclusions of this article will be made available by the authors, without undue reservation.

## Ethics statement

The study was approved by the Ethics Committee of Shanghai General Hospital, Shanghai Jiao Tong University School of Medicine. The patients/participants provided their written informed consent to participate in this study.

## Author contributions

Study design: N-GF and Y-DP; data collection: MT and X-HW; data analysis: MT and X-HW; data interpretation: N-GF and Y-DP; manuscript writing: all authors; final approval of manuscript: all authors.

## Funding

This study was supported by the National Natural Science Foundation of China (81870596, 82170827) and the Natural Science Foundation of Shanghai (21ZR1451200, 22ZR1450100).

## Conflict of interest

The authors declare that the research was conducted in the absence of any commercial or financial relationships that could be construed as a potential conflict of interest.

## Publisher’s note

All claims expressed in this article are solely those of the authors and do not necessarily represent those of their affiliated organizations, or those of the publisher, the editors and the reviewers. Any product that may be evaluated in this article, or claim that may be made by its manufacturer, is not guaranteed or endorsed by the publisher.
